# Effect of Deliberate Hypotension on Regional Cerebral Oxygen Saturation During Functional Endoscopic Sinus Surgery: A Randomized Controlled Trial

**DOI:** 10.3389/fsurg.2021.681471

**Published:** 2021-09-08

**Authors:** Ling Zhang, Yang Yu, Juan Xue, Weiping Lei, Yaqin Huang, Yong Li, Jianliang Sun

**Affiliations:** ^1^The Fourth Clinical Medical College, Zhejiang Chinese Medical University School of Medicine, Hangzhou, China; ^2^Department of Anesthesia, Hangzhou First People's Hospital Affiliated to Zhejiang University School of Medicine, Hangzhou, China

**Keywords:** functional endoscopic sinus surgery, deliberate hypotension, regional cerebral oxygen saturation, mean arterial pressure, nicardipine, esmolol

## Abstract

**Background:** Deliberate hypotension can reduce bleeding and improve visualization of the surgical field during functional endoscopic sinus surgery (FESS). However, hypotension may cause brain hypoperfusion and subsequent ischemic injuries, such as delayed awakening, stroke, postoperative delirium, and postoperative cognitive dysfunction. Near-infrared spectroscopy (NIRS) can be used to monitor real-time regional cerebral oxygen saturation (rSO_2_) levels to estimate brain perfusion. The present study aimed to evaluate the change in rSO_2_ induced by deliberate hypotension during FESS, and assess the impact of deliberate hypotension on the surgical process.

**Material and Methods:** A randomized controlled trial was registered with the Chinese clinical trial registry (ChiCTR2000039846). A total of 40 patients were enrolled and randomly divided into the control and intervention groups, and finally, 39 patients were analyzed. Deliberate hypotension was induced in the intervention group using nicardipine and esmolol, whereas the control group received general anesthesia without deliberate hypotension. We recorded mean arterial pressure (MAP), saturation of pulse oximetry (SpO_2_), rSO_2_, and heart rate (HR) before induction of anesthesia (T0), immediately after induction of anesthesia (T1), at the beginning of the operation (corresponding with the establishment of deliberate hypotension) (T2), 10 min (T3) and 20 min (T4) after the operation began, at the end of the operation (corresponding with the end of deliberate hypotension) (T5), and 5 min (T6) and 15 min (T7) after the operation. The partial pressure of end-tidal carbon dioxide (PetCO_2_) was recorded at T1, T2, T3, T4, T5, and T6. The duration of surgery, intraoperative blood loss, tracheal extubation time, and the number of patients that experienced cerebral desaturation events (CDEs) were recorded. The surgical field was estimated postoperation based on the Fromme score.

**Results:** A 30% decrease from the baseline MAP resulted in a decrease of intraoperative bleeding, improvement in the quality of the surgical field, and the shortening of the duration of surgery during FESS in the intervention group compared with the control group. In addition, rSO_2_ was reduced and no CDEs were experienced in the intervention group. Linear regression analysis demonstrated a correlation between the decline in rSO_2_ and that in MAP.

**Conclusions:** A decrease in MAP to a certain level will cause a decrease of rSO_2_ in patients undergoing FESS under general anesthesia. Based on our findings, we recommend that the deliberate hypotensive target indicated by MAP be reduced by 30%, while PetCO_2_ is maintained at 35–40 mmHg and HR is maintained at about 60 beats per minute during FESS.

## Introduction

With the development of nasal visualization technology, functional endoscopic sinus surgery (FESS) has become the gold standard for treating medically refractory chronic sinusitis. Given the sensitive anatomical structures in the narrow spaces of the nasal cavities, such as the carotid artery, anterior ethmoidal artery, orbit, optic nerve, and intracranial contents of the anterior cranial fossa just beyond the skull base, even a small visible bleed into the surgical field may affect the surgical field of vision, thereby causing tissue damage and serious complications (1, 2). Thus, an optimal surgical field of view is crucial for adequate visualization and identification of sensitive neurovascular structures (2).

At present, various pre- and intraoperative strategies can be used to reduce or prevent bleeding into the surgical field and improve visualization, such as the decreasing of the mean arterial pressure (MAP), lowering heart rate (HR), local injection of vasoconstrictors, application of topical sinonasal vasoconstrictors, preoperative use of steroids, reversal of the Trendelenburg position, and induction of deliberate hypotension with anesthesia (3). Deliberate hypotension is defined as the reduction of systolic blood pressure (BP) to 80–90 mmHg, and that of MAP to 50–65 mmHg or 30% lower than the baseline level (4). However, extreme hypotension may cause organ insufficiency and subsequent ischemic injury of vital organs, especially the brain (1).

Near-infrared spectroscopy (NIRS) is a non-invasive method of monitoring regional cerebral oxygen saturation (rSO_2_) in real time (5) that allows estimation of brain perfusion and early detection of cerebral desaturation events (CDEs), which is defined as a >20% reduction in rSO_2_ for at least 15 s or <55% drop in its absolute level (5). NIRS can diagnose insufficient oxygen saturation on time, and prevent hypoxemia and other complications of anesthesia (6). Cerebral oximetry was first used for intraoperative monitoring during cardiac surgery to evaluate cerebral perfusion (7). It has been gradually adopted in non-cardiac operations as well, such as thoracic surgery and vascular surgery (7). Recent reports suggest that NIRS can guide deliberate hypotension by monitoring cerebral perfusion during FESS (2, 8). However, these studies are only prospective observational studies.

The present study aimed to conduct a randomized controlled trial (RCT) to evaluate the change in rSO_2_ induced by deliberate hypotension during FESS, and assess the impact of deliberate hypotension on surgery.

## Materials and Methods

### Patients

The RCT was conducted at the Hangzhou First People's Hospital affiliated with Zhejiang University School of Medicine following approval by the Ethics Committee (2020YLSD002-01) and was registered with the Chinese clinical trial registry (ChiCTR2000039846). A total of 40 patients who were diagnosed with chronic rhinosinusitis and scheduled for FESS were enrolled in this study and all participants signed the informed consent form. The inclusion criteria were as follows: (1) patients with an American Society of Anesthesiologists (ASA) class I and II status; (2) basal BP ≤140/90 mmHg; and (3) BP of hypertensive patients with antihypertensive agents ≤140/90 mmHg. The exclusion criteria were as follows: (1) hemoglobin (Hb) levels <100 g/L; (2) preoperative mini-mental state examination (MMSE) score ≤23 points; (3) cerebrovascular disease; (4) anxiety and mental disorders; (5) uncontrolled hypertension; and (6) coagulopathy and use of platelet inhibitors or anticoagulant therapy.

The patients were randomly assigned to the control and intervention groups (*n* = 20 each) using a list of numbers generated by the QuickCalcs (GraphpadPrism 7). The group assignment numbers were sealed in an envelope, and opened once written informed consent was obtained. The groups were homogenous in terms of age, gender, weight, Hb levels, baseline MAP and rSO_2_, and preoperative MMSE scores.

### Experimental Protocols

The patients were instructed to fast before the operation as per standard guidelines. ECG, HR, non-invasive BP, saturation of pulse oximetry (SpO_2_), temperature, and bispectral index (BIS) were measured in the operating room with the patient in supine position. Then, the right radial artery was punctured and catheterized to monitor invasive arterial BP. NIRS sensors (FORE-SIGHT oximeter, CASMED) were placed on both the right and left sides of the forehead before the induction of anesthesia to monitor rSO_2_. The baseline right and left rSO_2_ were measured before anesthesia induction when the patient breathed the room air, and the mean was calculated. Midazolam (0.04 mg/kg) was injected 5 min before the induction of anesthesia, and the baseline MAP was recorded 5 min later. Anesthesia was induced with propofol (1 mg/kg), etomidate (0.2 mg/kg), sufentanil (0.5 μg/kg), and cisatracurium (0.2 mg/kg), and tracheal intubation was performed. Volume-controlled ventilation was provided with tidal volume 6–8 ml/kg, the fraction of inspired oxygen maintained at 50% in an oxygen air mixture, with a respiration rate 12–15 times per minute. The respiration rate and tidal volume were adjusted according to the partial pressure of end-tidal carbon dioxide (PetCO_2_) of 35–40 mmHg. BIS was maintained between 40 and 60 with a continuous infusion of propofol and remifentanil, and continuous inhalation of 1–1.5% sevoflurane. A total of 2 ml lignocaine-adrenaline (1:2,00,000) was injected into the operative region at the beginning of the operation.

Before surgery, hypotension was induced immediately by reducing MAP to 70% of the baseline and was maintained by continuous infusion of nicardipine plus esmolol in the intervention group (nicardipine 20 mg plus esmolol 200 mg diluted 0.9% saline solution to 50 ml and adjusting infusion rate by MAP level). The control group received only general anesthesia without deliberate hypotension. In case CDEs were encountered, nicardipine and esmolol infusion was stopped, anesthetic drugs were adjusted, and the patients were given fluid therapy and 5 mg intravenous ephedrine if needed.

At the ending of the operation, all drugs were discontinued. The tracheal catheter was removed when the patient regained spontaneous breathing and consciousness, recovered cough and swallowing reflex, the patient could lift his/her head off the pillow for more than 5 s, the breathing rate was 10–20 times/min and SpO_2_ > 95% with breathing air.

Mean arterial pressure, SpO_2_, HR, and rSO_2_ were recorded before induction of anesthesia (T0), immediately after induction of anesthesia (T1), at the beginning of the operation (which corresponded to the establishment of deliberate hypotension) (T2), 10 min (T3) and 20 min (T4) after the operation began, at the end of the operation (which corresponded to the end of deliberate hypotension) (T5), and finally 5 min (T6) and 15 min (T7) after the end of the operation. PetCO_2_ was recorded at T1, T2, T3, T4, T5, and T6. The duration of surgery (the duration of deliberate hypotension), intraoperative blood loss, tracheal extubation time, and the number of patients that experienced CDEs were recorded.

After the surgery, the surgical field was evaluated by the surgeon using Fromme score, which is as follows: 1—slight bleeding without the requirement for blood suctioning, 2—slight bleeding requiring occasional suctioning but surgical field not compromised, 3—slight bleeding requiring frequent suctioning and surgical field affected within a few seconds of removing suction, 4—moderate bleeding requiring frequent suctioning and surgical field directly compromised by bleeding after suction is removed, and 5—severe bleeding that overwhelmed constant suctioning and severely compromised the surgical field and prevented surgery (9).

Cognitive function was tested on the evening before surgery was performed and 1 day after surgery using the MMSE scale.

### The INVOS System

FORE-SIGHT oximeter (CASMED) is a two-channel probe that allows continuous, non-invasive, and real-time measurement of cerebral oxygenation. The FORE-SIGHT oximeter is a two-channel (right and left) NIRS cerebral oximeter that automatically analyzes and calculates the rSO_2_ value detected by sensors.

### Statistical Analysis

The sample size for calculating the difference in rSO_2_ between the two groups during deliberate hypotension was determined using G-power (version 3.1.9.2, α = 0.05, power = 0.8) depending on our preliminary study results (intervention group: mean = 70.83, SD = 3.041; control group: mean = 73.5, SD =2.437, effect size = 0.969). A total of 18 patients were required per group, and considering an expulsion rate of 0.2, the sample size was increased to 20 patients per group.

All variables were expressed as numbers, mean (SD) or median (interquartile range). Statistical analysis was performed using the GraphPad Software (Prism7 for Mac OSX). Normally distributed continuous data were compared using the unpaired *t*-test, and the Mann–Whitney *U*-test was used for data with non-normal distribution. Two-way ANOVA was used to analyze serial changes in HR, MAP, PetCO_2_, and rSO_2_ data with time using the intragroup and group values as interfactor measurements. Spearman's correlation analysis was used to analyze the relationship between percentage decrease in rSO_2_ and MAP. Categorical variables were analyzed using Pearson's chi-square test or Fisher's exact test. *P* < 0.05 was considered to indicate statistical significance.

## Results

**Figure 1:**
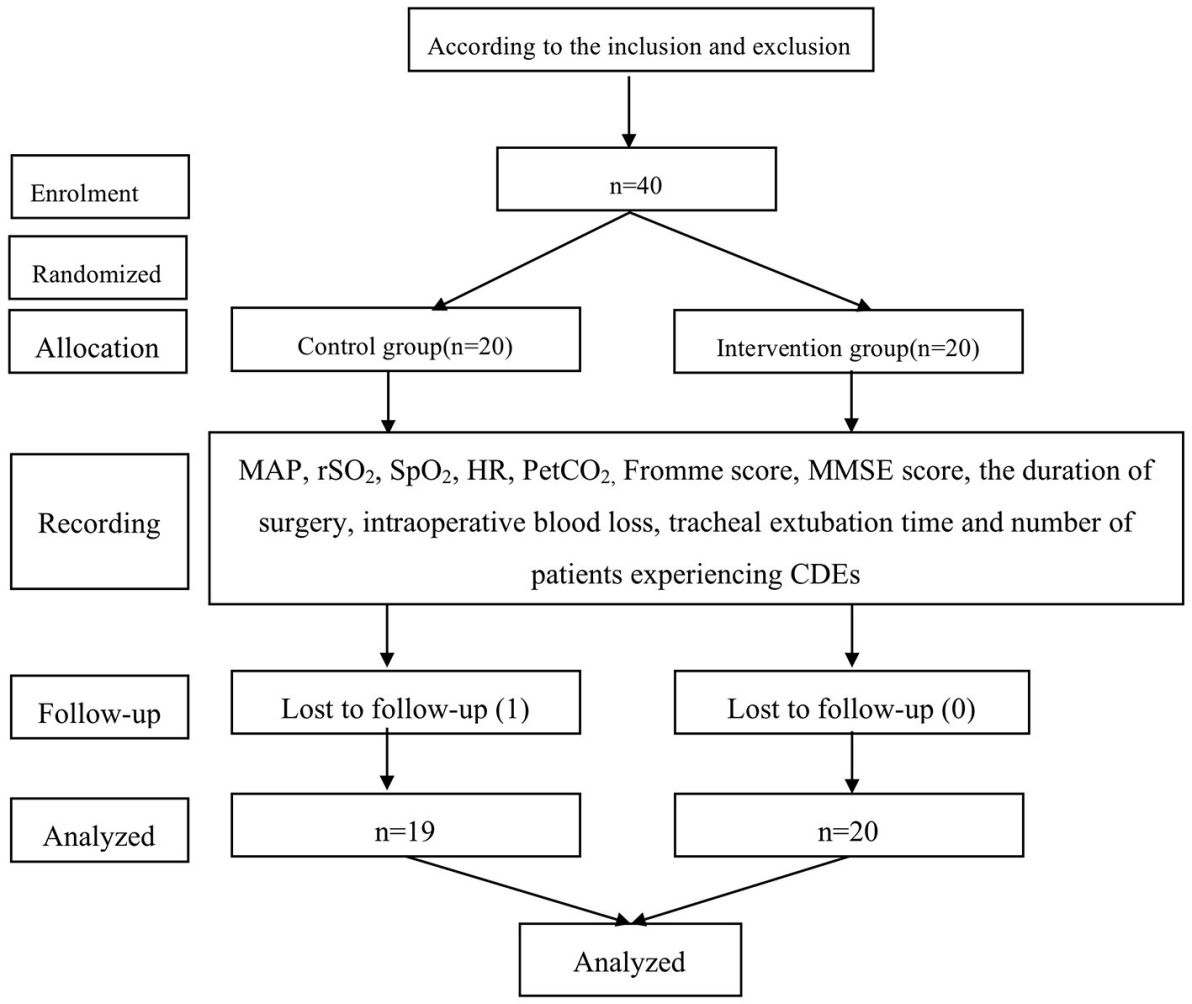
Study flowchart.

**Figure 2:**
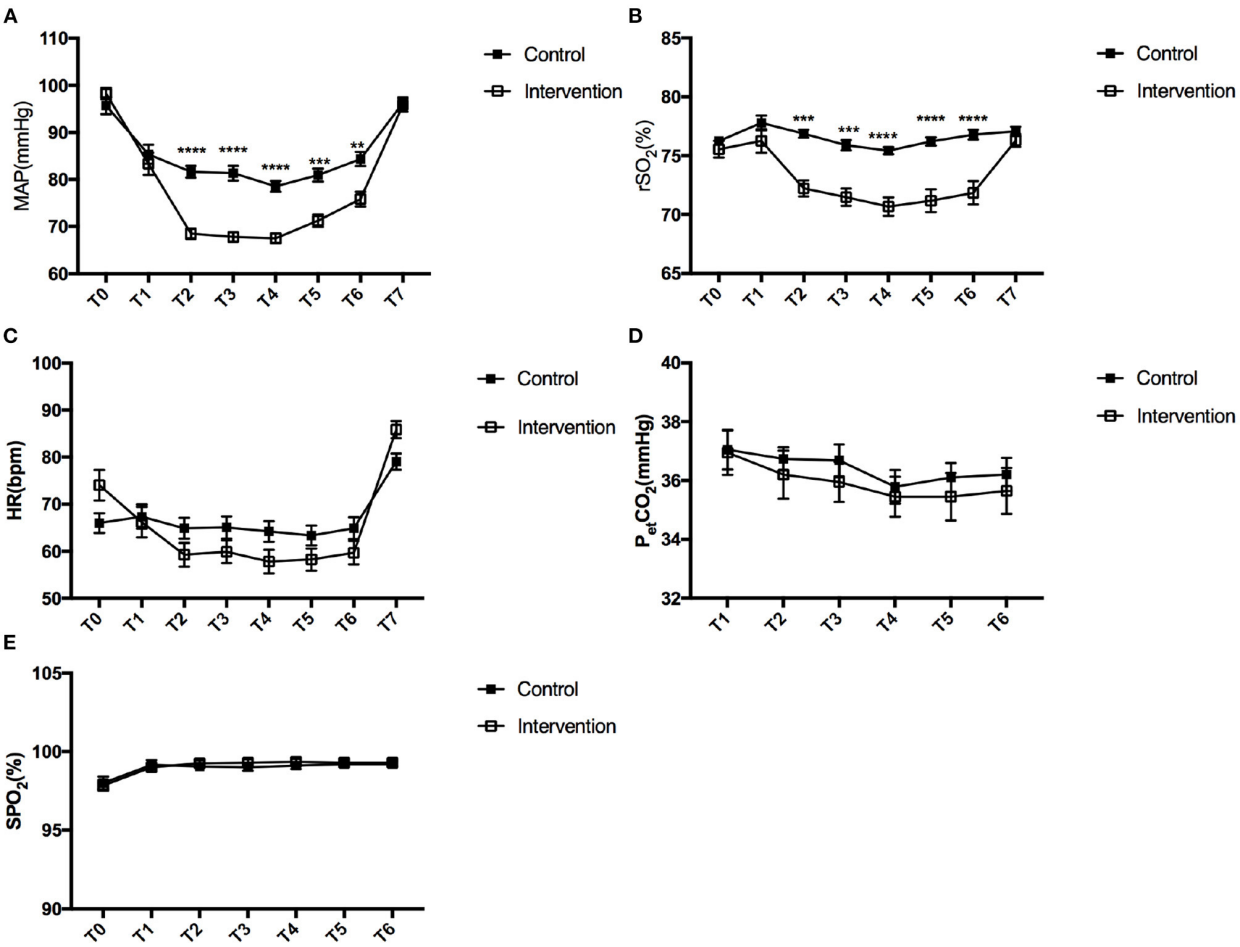
Change in MAP **(A)**, rSO_2_ **(B)**, HR **(C)**, PetCO_2_ **(D)**, and SpO_2_ **(E)** between the control and intervention groups during functional endoscopic sinus surgery. A square with error bars represents the mean and standard error of mean. T0 (baseline), before induction of anesthesia; T1, immediately after induction of anesthesia; T2, at the beginning of the operation; T3, 10 min after the operation began; T4, 20 min after the operation began; T5, at the end of the operation; T6, 5 min after the end of the operation; T5, 15 min after the end of the operation. MAP, mean arterial pressure; rSO_2_, regional cerebral oxygen saturation; HR, heart rate; SpO_2_, peripheral oxygen saturation; PetCO_2_, partial pressure of end-tidal carbon dioxide. **P < 0.01, ***P < 0.001, ****P < 0.0001 vs. control group.

Based on the inclusion and exclusion criteria, 40 patients were initially enrolled in this study, and one patient was later excluded due to loss of data (Figure 1). The demographic data of the remaining 39 patients are shown in Table 1, which indicates a lack of any significant difference between the control and intervention groups in terms of age, gender, weight, Hb levels, history of hypertension, and ASA physical status (*P* > 0.05). Table 2 shows the results of the variables monitored during surgery. Duration of surgery, Fromme score, and intraoperative blood loss in the intervention group were lower than that of the control group (*P* < 0.05). However, the tracheal extubation time in the intervention group was longer, compared with the control group (*P* < 0.05). The changes in MAP during FESS are shown in Table 3; Figure 2A. There was no difference in the baseline MAP values at T0 (*P* > 0.05). The MAP values at T1, T2, T3, T4, T5, and T6 were lower than that at T0 (baseline) in both the control and intervention groups (*P* < 0.05). However, the MAP values were restored to the baseline level at T7 (*P* > 0.05). Compared with the control group, the MAP values were lower in the intervention group at T2, T3, T4, and T5 (*P* < 0.05).

**Table 1:** Demographic and preoperative characteristics.

**Variable**	**Control (*n* = 19)**	**Intervention (*n* = 20)**	***P*-value**
Age (years)	48 ± 13.86	50.55 ± 14.34	0.576
Gender (male/female)	13/6	12/8	0.741
ASA physical status (I/II)	1/18	3/17	0.605
Weight (kg)	66 ± 10.86	63.1 ± 10.59	0.404
Hypertension, *n*	5	4	0.716
Hemoglobin (g/l)	146.50 ± 13.71	143.10 ± 16.76	0.491

*All values are given as mean ± SD or number of patients. ASA, American Society of Anesthesiologists*.

**Table 2:** Clinical characteristics of control and intervention groups.

**Variable**	**Control (*n* = 19)**	**Intervention (*n* = 20)**	***P*-value**
Duration of surgery (min)	93.11 ± 33	71.85 ±29.36^*^	0.040
The tracheal extubation time (min)	8.79 ±3.54	11.80 ± 4.94^*^	0.036
Fromme score	3.37 ± 0.60	2.80 ± 0.52^**^	0.003
Intraoperative blood loss (mL)	77(55–100)	47(30–101.3)^*^	0.034

*All values are given as mean ± SD or median (interquartile range)*.

* *P < 0.05*,

** *P < 0.01*.

**Table 3:** Comparison of MAP (mmHg) at each time point between control and intervention groups.

**Groups**	**T0**	**T1**	**T2**	**T3**	**T4**	**T5**	**T6**	**T7**
**Control (** ***n*** **=** **19)**	95.68 ± 7.83	85.37 ± 8.83^###^	81.63 ± 5.43^####^	81.34 ± 6.81^####^	78.55 ± 4.98^####^	80.95 ± 0.04^####^	84.37 ± 6.60^####^	96.26 ± 5.24
**Intervention (** ***n*** **=** **20)**	98.25 ± 5.57	83.35 ± 10.52^####^	68.50 ± 4.92^****^^####^	67.80 ± 4.58^****^^####^	67.50 ± 4.70^****^^####^	71.25 ± 5.85^***^^####^	75.85 ± 7.00^*^^####^	95.80 ± 6.08

*All values are given as mean ± SD*.

** *P < 0.01;*

*** *P < 0.001;*

**** *P < 0.0001 vs. control group*.

### *P < 0.001;*

#### *P < 0.001 vs. T0. T0 (baseline), before induction of anesthesia; T1, immediately after induction of anesthesia; T2, at the beginning of the operation; T3, 10 min after the operation began; T4, 20 min after the operation began; T5, at the end of the operation; T6, 5 min after the end of the operation; T5, 15 min after the end of the operation. MAP, mean arterial pressure*.

Changes in rSO_2_ are shown in Table 4; Figure 2B during FESS. There was no difference in the baseline rSO_2_ values at T0 (*P* > 0.05) and the baseline rSO_2_ value was 75.89% in our study. The rSO_2_ values at T2, T3, T4, T5, and T6 were lower than that at T0 (baseline) in the intervention group (*P* < 0.05), while the value was restored to the baseline level at T7 (*P* > 0.05). Unlike the pattern of the MAP values, there was no additional decrease in rSO_2_ values in the control group (*P* > 0.05). In both groups, the rSO_2_ values at T1 were not lower than that at T0 (*P* > 0.05). Compared with the control group, the rSO_2_ values were significantly lower in the intervention group at T2, T3, T4, T5, and T6 (*P* < 0.05). However, there was no significant difference at T1 and T7 (*P* > 0.05).

**Table 4:** Comparison of rSO_2_ (%) at each time point between control and intervention groups.

**Groups**	**T0**	**T1**	**T2**	**T3**	**T4**	**T5**	**T6**	**T7**
Control (*n* = 19)	76.24 ± 1.50	77.79 ± 2.73	76.87 ± 1.49	75.90 ± 1.88	75.42 ± 1.33	76.21 ± 1.55	76.79 ± 1.84	77.08 ± 1.69
Intervention (*n* = 20)	75.55 ± 3.13	76.25 ± 4.54	72.23 ± 3.04^***#^	71.48 ± 3.28^***^^##^	70.68 ± 3.51^****^^####^	71.18 ± 4.34^****^^###^	71.85 ± 4.45^****^^##^	76.3 ± 2.45

*All values are given as mean ± SD*.

*** *P < 0.001;*

**** *P < 0.0001 vs. control group*.

# *P < 0.05;*

## *P < 0.01;*

### *P < 0.001l;*

#### *P < 0.001 vs. T0. T0 (baseline), before induction of anesthesia; T1, immediately after induction of anesthesia; T2, at the beginning of the operation; T3, 10 min after the operation began; T4, 20 min after the operation began; T5, at the end of the operation; T6, 5 min after the end of the operation; T5, 15 min after the end of the operation. rSO_2_, regional cerebral oxygen saturation*.

**Figure 3:**
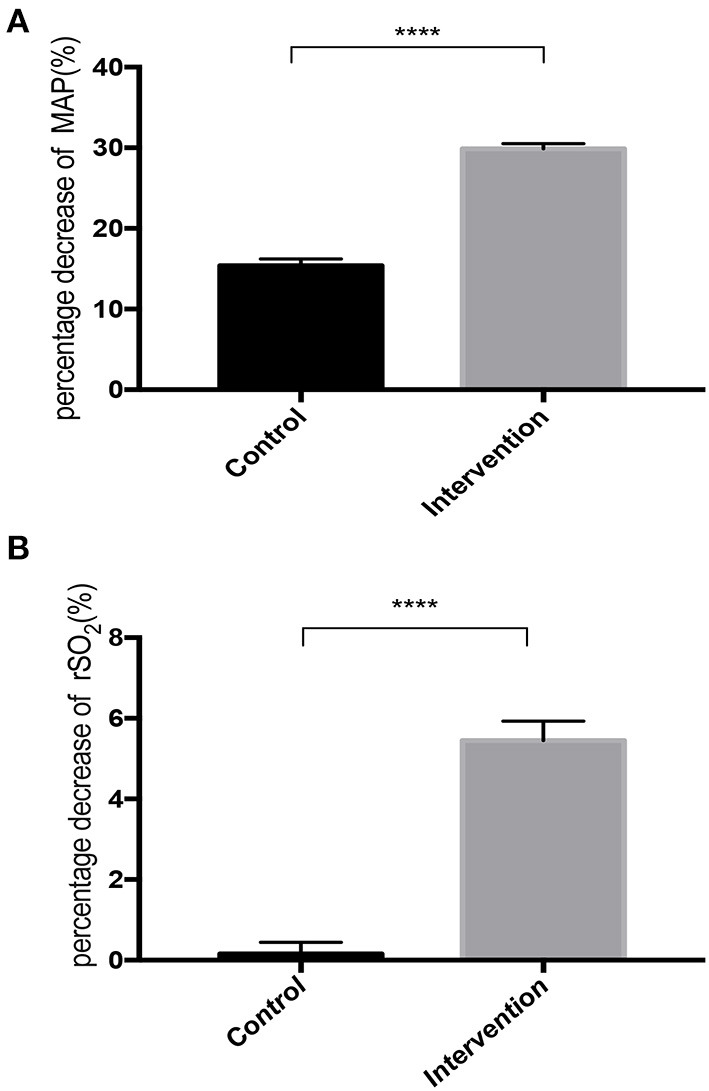
Percentage decrease in MAP **(A)** and rSO_2_ **(B)** during deliberate hypotension in control and intervention groups. Filled square with error bars represent the mean and SE of mean. ****P < 0.0001 vs. control group.

The decline in cognitive function on day 1 postoperative was measured using MMSE but was not observed in any of the patients (*P* > 0.05) (Table 5). Figures 2C–E shows that the intraoperative parameters, HR, PetCO_2_, and SpO_2_, at each recording point were similar between these groups (*P* > 0.05). Figure 3 shows the decreases in MAP and rSO_2_ from the baseline in the deliberate hypotension group (*P* < 0.05). When MAP decreased by ~30%, the percentage decrease of rSO_2_ from the baseline was ~5% in the intervention group.

**Table 5:** Comparison of preoperative and postoperative MMSE scores.

	**Control (*n* = 19)**	**Intervention (*n* = 20)**	***P*-value**
Preoperative	28.58 ± 0.86	28.85 ± 0.99	0.873
Postoperative first day	28.73 ± 0.93	28.50 ± 1.10	0.885

*Data are presented as mean ± SD. MMSE, mini-mental state examination*.

Analysis of all patients, irrespective of the group they belonged to, demonstrated a significant correlation between the percentage decrease in MAP and the percentage decrease in rSO_2_ (*r* = 0.627, 95% CI: 0.546–0.695).

## Discussion

In this study, we observed that a 30% decrease of baseline MAP resulted in decreased intraoperative bleeding, improved quality of the surgical field, and resulted in a shorter duration of surgery during FESS. More importantly, although rSO_2_ values decreased, none of the patients experienced CDEs. Correlation analysis of the percentage of decrease of rSO_2_ and MAP demonstrated a correlation between the decline of rSO_2_ and the level of decrease of MAP. At the same time, analysis of cognitive function on day 1 postoperation showed that postoperative cognitive dysfunction (POCD) caused by MMSE was not observed in any of the patients.

Effective hemostasis is crucial during FESS since even minor bleeding can severely compromise an already restricted view in the narrow nasal space, prolong the operation, and increase the risk of damage to sensitive tissue structures. The lowering of MAP during general anesthesia can minimize intraoperative bleeding, reduce the time of operation, and improve the quality of surgery (3, 10). A prospective, observational cohort study showed that MAP below 60 mmHg was associated with better surgical visibility in terms of bleeding assessment score (1). Consistent with this, we found that the quality of surgical field was enhanced in the intervention group compared to the control group in terms of Fromme score, which indicates less bleeding and a shorter duration of surgery.

However, a lower MAP can decrease cerebral blood flow (CBF) and thus increase the risk of cerebral hypoperfusion, eventually leading to postoperative neurological injuries, such as stroke, postoperative delirium (POD), and POCD (11). A large retrospective cohort analysis of 7,457 patients undergoing cardiac surgery showed a strong association between continuous MAP below 64 mmHg and postoperative stroke during cardiopulmonary bypass, and the severity and duration of hypotension were closely connected with the risk of stroke after surgery (12). Another case-control study of non-cardiac and non-neurosurgical procedures showed that the duration of baseline MAP reduction by 30% was linked with stroke within 10 days postsurgery (13). Similarly, a retrospective study conducted on 1,083 patients after general anesthesia showed that 35% of patients suffered from delirium after surgery, and perioperative hypotension was moderately correlated with higher odds of postoperative delirium (14).

Cerebral pressure autoregulation, MAP between 60 and 150 mmHg, maintains a relatively stable CBF. However, cerebral autoregulation is subject to variation, and its plateau, lower and upper limits are affected by age, gender, diseases, metabolic rate, vasoactive drugs, volatile anesthetics, sympathetic tone, Hb and oxygen content, and CO_2_ levels (15). Continuous monitoring of rSO_2_ using the NIRS can reflect the oxygen supply, oxygen consumption, and cerebral perfusion on time (16). Studies have shown that decreased intraoperative rSO_2_ is significantly associated with postoperative neurological dysfunction (17–19). A prospective cohort study including 43 patients undergoing cerebral endovascular surgery showed that the decrease of rSO_2_ can be used to predict delirium and that the continuous monitoring of rSO_2_ can reduce the incidence of postoperative delirium (19). Another study revealed that the duration of decline in rSO_2_ < 60% during lumbar spinal surgery was correlated with the development of POCD (17). Therefore, monitoring of rSO_2_ and timely intervention can reduce the occurrence of postoperative neurological disorders. The criteria for cerebral ischemia are reduction of 10 index points in rSO_2_ from a stable baseline, absolute value of rSO_2_ < 50%, 20–25% reduction in relative rSO_2_, and >25% difference between the left and right side rSO_2_ (7). Thus, a 20% drop in rSO_2_ from the baseline was used as the threshold in this study, and deliberate hypotension was stopped in the event of further drop till rSO_2_ was restored to acceptable levels.

We surveyed the effect of controlled hypotension on rSO_2_ during FESS. Our results indicated a correlation between the decline in rSO_2_ and the decrease in MAP (*r* = 0.627, *P* < 0.0001). An observational study of 41 patients undergoing FESS found that MAP was moderately cross-correlated with current rSO_2_ (*r* = 0.728) (2). At the same time, the correlation analysis between PetCO_2_ and rSO_2_ also found a correlation between them (2). However, a prospective, blinded, observational trial conducted on 31 healthy patients showed that cerebral oximetry was directly correlated with PetCO_2_ but not with MAP during sinus endoscopy (8). CO_2_ levels can influence CBF by regulating the dilation and constriction of cerebral vessels and alter rSO_2_ levels (20). Therefore, we kept PetCO_2_ between the control and intervention groups at a similar level. In our study, carbon dioxide was found to be an essential factor that affected rSO_2_ control. Thus, our results show that a reduction in rSO_2_ is related to the level of decrease in MAP. Previously published results have shown that the rSO_2_ value decreased when patients were placed in the beach-chair position, then induced hypotension did not lead to a reduction of the rSO_2_ value while undergoing arthroscopic shoulder surgery (21). Therefore, when other major factors that affect rSO_2_ are controlled, the rSO_2_ value is directly correlated with MAP.

Our results showed that there was no difference between baseline MAP and rSO_2_ values, and that the baseline rSO_2_ value was 75.89% in our study population. The MAP values decreased in both groups immediately after induction of anesthesia (T1), but the value of rSO_2_ did not show a corresponding decline. This may be related to the inhalation of 100% oxygen during anesthesia induction. The time point that corresponded to the period of deliberate hypotension was T2 (at the beginning of the operation), T3 (10 min after the operation began), T4 (20 min after the operation began), and T5 (at the end of the operation). During deliberate hypotension, the rSO_2_ values were lower in the intervention group compared with the control group. When baseline MAP decreased by ~30%, the decrease in rSO_2_ from the baseline was only ~5% in the intervention group. In the control group, when the baseline MAP decreased as a result of the use of anesthesia, no additional decrease in rSO_2_ value was found. Since the reduction from baseline MAP did not proceed beyond the range of cerebral autoregulation. Both MAP and rSO_2_ values were restored to baseline levels 15 min after the end of the operation.

Several anesthetics and vasoactive agents have been used to establish controlled hypotension. In our study, the combined use of nicardipine and esmolol decreased the MAP to 70% of the baseline and maintained it at this level. Nicardipine is a calcium channel antagonist that induces hypotension and exerts a protective effect on renal function (22). It is also known to cause tachycardia (22) which increases myocardial metabolism and shortens diastole, thereby reducing myocardial perfusion. Esmolol is a β-blocker that can inhibit reflex tachycardia, decrease myocardial oxygen consumption, reduce cardiac output (CO), and finally lower the MAP (23). Sun et al. demonstrated that deliberate hypotension using nicardipine combined with esmolol not only maintained hemodynamic stability and decreased perioperative stress response, but also reduced the incidence of POCD in elderly patients (24). Therefore, in our study, continuous infusion of nicardipine plus esmolol was used to maintain MAP at 70% of the baseline in the intervention group. Our investigation showed that HR was relatively stable during deliberate hypotension using nicardipine plus esmolol, and that there was no significant difference between the control and intervention groups. Nicardipine plus esmolol may be a better choice to induce deliberate hypotension during FESS.

During deliberate hypotension, the reduction of MAP will lead to cerebral hypoperfusion and hypoxia, and postoperative neurological injury. In our study, we analyzed the postoperative cognitive function and did not observe any decline in either group. Furthermore, none of the patients experienced CDEs when the baseline MAP was decreased by 30%. Studies have shown that patients undergoing FESS with deliberate hypotension (>75%, 65–75%, and <65% of baseline) seemed to be equally safe for the patient as anesthetic management in normotension, simultaneously decreasing the complication rate, such as renal, lung, cardiovascular system damage, and postoperative cognitive decline (25).

This study has some limitations. First, we only analyzed the cognitive function on the first day postsurgery, do not evaluate the next longer period of cognitive function and other postoperative neurological injury, such as stroke and POD. Second, the level of biomarkers of cerebral ischemic change, such as S-100ß and glial fibrillary acidic protein were also not tested. Third, postoperative adverse reactions, such as nausea and vomiting were not compared between the two groups of patients.

## Conclusion

A decrease in MAP up to a certain level will decrease rSO_2_ in patients undergoing FESS under general anesthesia. Our results showed that when the baseline MAP was reduced by 30%, the decrease in rSO_2_ from the baseline was only ~5%, which is still within safe levels. The reduction in rSO_2_ is particularly associated with the decrease of MAP. Based on our findings, we recommend that the deliberate hypotensive target indicated by MAP be reduced by 30%, while PetCO_2_ be maintained at 35–40 mmHg and HR be maintained at about 60 beats per minute during FESS, which not only decreases intraoperative bleeding, improves the quality of the surgical field and reduces the duration of surgery during FESS, but also avoids the occurrence of CDEs. Therefore, nicardipine plus esmolol may be a better choice to induce deliberate hypotension during FESS.

## Data Availability Statement

The original contributions presented in the study are included in the article/supplementary material, further inquiries can be directed to the corresponding author/s.

## Ethics Statement

The studies involving human participants were reviewed and approved by Hangzhou First People's Hospital Affiliated to Zhejiang University school of Medicine. The patients/participants provided their written informed consent to participate in this study.

## Author Contributions

LZ and JS: conception and design. JS: administrative support and randomization and maintaining the group assignment numbers. YY and WL: anesthesia and deliberate hypotension. JX and YH: collection of data. YL: fromme score. LZ: data analysis. All authors wrote and approved the manuscript.

## Funding

The project was supported by Zhejiang University of Traditional Chinese Medicine Postgraduate Top-notch Innovative Talent Cultivation Program (ZHYD2017-17), The Key Project of Joint Pre-research Fund (YYJJ2019Z03) for Clinical Scientific Research of Hangzhou First People's Hospital Affiliated to Zhejiang University, Medical Scientific Research Foundation of Zhejiang province (2015ZYC-A34), and the Natural Science Foundation of Zhejiang Province (LY21H090005).

## Conflict of Interest

The authors declare that the research was conducted in the absence of any commercial or financial relationships that could be construed as a potential conflict of interest.

## Publisher's Note

All claims expressed in this article are solely those of the authors and do not necessarily represent those of their affiliated organizations, or those of the publisher, the editors and the reviewers. Any product that may be evaluated in this article, or claim that may be made by its manufacturer, is not guaranteed or endorsed by the publisher.
